# Comprehensive Fungal Community Analysis of House Dust Using Next-Generation Sequencing

**DOI:** 10.3390/ijerph17165842

**Published:** 2020-08-12

**Authors:** Kazuki Izawa, Atsutaka Kubosaki, Naoki Kobayashi, Yutaka Akiyama, Akiko Yamazaki, Kazuhiro Hashimoto, Rumi Konuma, Yoichi Kamata, Yukiko Hara-Kudo, Kenichi Hasegawa, Toshiharu Ikaga, Maiko Watanabe

**Affiliations:** 1 Department of Computer Science, School of Computing, Tokyo Institute of Technology, Meguro-ku, Tokyo 152-8550, Japan; izawa@bi.c.titech.ac.jp (K.I.); akiyama@c.titech.ac.jp (Y.A.); 2 Division of Microbiology, National Institute of Health Sciences, Kawasaki-ku, Kawasaki, Kanagawa 210-9501, Japan; kubosaki@nihs.go.jp (A.K.); ykudo@nihs.go.jp (Y.H.-K.); 3 Department of Food and Life Science, School of Life and Environmental Science, Azabu University, Chuo-ku, Sagamihara, Kanagawa 252-5201, Japan; n-kobayashi@azabu-u.ac.jp; 4 Department of Veterinary Medicine, Faculty of Agriculture, Iwate University, Morioka, Iwate 020-8550, Japan; ayamazak@iwate-u.ac.jp; 5 Laboratory of Integrated Pest Management, FCG Research Institute, Inc., Koto-ku, Tokyo 135-0064, Japan; hashimoto@fcg-r.co.jp; 6 Tokyo Metropolitan Industrial Technology Research Institute, Koto-ku, Tokyo 135-0064, Japan; konuma.rumi@iri-tokyo.jp; 7 Department of Food Design, Faculty of Nutritional Science, Koshien University, Takarazuka, Hyogo 665-0006, Japan; y-kamata@cs.kinran.ac.jp; 8 Department of Food and Nutrition, Faculty of Human Life Science, Senri Kinran University, Suita, Osaka 565-0873, Japan; 9 Department of Architecture and Environment Systems, Faculty of Systems Science and Technology, Akita Prefectural University, Yurihonjo, Akita 015-0055, Japan; haseken@akita-pu.ac.jp; 10 Department of System Design Engineering, Faculty of Science and Technology, Keio University, Kohoku-ku, Yokohama, Kanagawa 223-8522, Japan; ikaga@sd.keio.ac.jp

**Keywords:** house dust, fungal community analysis, ITS region, next-generation sequencing

## Abstract

Fungal community analyses in homes have been attracting attention because fungi are now generally considered to be allergens. Currently, these analyses are generally conducted using the culture method, although fungal communities in households often contain species that are difficult to culture. In contrast, next-generation sequencing (NGS) represents a comprehensive, labor- and time-saving approach that can facilitate species identification. However, the reliability of the NGS method has not been compared to that of the culture method. In this study, in an attempt to demonstrate the reliability of this application, we used the NGS method to target the internal transcribed spacer 1 (ITS1) in the fungal genome, conducted fungal community analyses for 18 house-dust samples and analyzed fungal community structures. The NGS method positively correlated with the culture method regarding the relative abundance of *Aspergillus*, *Penicillium*, *Cladosporium* and yeasts, which represent the major fungal components found in houses. Furthermore, several genera, such as *Malassezia*, could be sensitively detected. Our results imply that the reliability of the NGS method is comparable to that of the culture method and indicates that easily available databases may require modifications, including the removal of registrations that have not been sufficiently classified at the genus level.

## 1. Introduction

To identify the members of the microbial community in an environment, a commonly used method is the culture method. However, because of the selectiveness of various media, conducting a comprehensive survey of microbial communities can be difficult by using this method. In contrast, molecular-based methods, such as quantitative-PCR [1], PCR-denaturing gradient gel electrophoresis [2] and next-generation sequencing (NGS), can reveal more comprehensive information regarding the contents of a microbial community. The development of NGS has made it possible to apply a metagenomic approach as the most powerful DNA-sequencing-based method for the comprehensive detection of microbial community structures in various environments [3,4].

Fungal community analyses in households have begun to attract attention because fungi are now generally considered to be allergens [5,6]. Many researchers have previously reported cases of patients with asthma, allergic bronchopulmonary mycosis (ABPM) and other illnesses caused by fungal products [7,8]. Currently, the culture method remains the primary approach used to conduct fungal community analysis for dwellings [9,10,11] and the application of molecular methods, including the NGS method, remains limited because the sequence registrations for fungi in databases are incomplete, and the procedures and methods used to identify fungal sequences have not yet been standardized [12]. However, the culture method has three primary limitations. First, the culture method can only detect limited taxa because some fungi have failed to be cultured on any media, resulting in the inability to provide a truly comprehensive survey of fungal populations. Second, the culture method is time- and labor-intensive. To obtain an easily and accurately countable colony, researchers must prepare serial dilutions for each test solution and spread these onto many agar plates. Additionally, researchers must wait until colonies appear on the plates, which can take seven days or longer. Third, once fungal colonies have appeared on the agar plates, they must be identified, which requires many experiments to be conducted, and researchers require significant experience and knowledge to accurately identify fungal species. Therefore, new procedures and methods that address these three limitations in the culture method and are suitable for fungal community analyses in households must be established.

Here, to address the limitations of the culture method, we demonstrate the application of the NGS method to conduct fungal community analysis of household dust.

## 2. Materials and Methods

### 2.1. Sample Collection

We collected house dust samples from bedroom floors of detached houses using a vacuum cleaner (Makita Corporation, Aichi, Japan) between December 2016 and March 2017. We sampled 18 houses, which were located in two specific regions among eight divisions in Japan, which are defined based on the average temperature and in accordance with the building energy efficiency act. Moreover, we investigated the flooring materials and the floor area. We separated large foreign particles from the collected house dust and cut these components into small pieces to ensure the total dust samples were evenly distributed. Then, we measured the weight of each total dust sample. The dust samples were then homogenized using a stomacher with nine-fold phosphate-buffered saline (PBS) according to the weight of the house dust sample, and the homogenates were serially diluted (*w/w*) from 10^1^ to 10^6^ in PBS to generate test solutions for the culture method, as described below.

### 2.2. Culture Method for the Isolation of Fungi

The surface plating method was used to enumerate the fungi in house dust according to a standard protocol [13]. Five 200-µL aliquots of each homogenate and their respective serial dilutions were spread onto dichloran–glycerol 18 agar (DG-18 agar; Thermo Fisher Scientific, Inc., Waltham, MA, USA) plates supplemented with chloramphenicol (100 mg/L). The cultures were plated in triplicate and incubated at 25 °C for 7 days. Fungal colonies on the agar plates were counted separately based on the macroscopic and microscopic features of each colony, and viable fungal counts were expressed as colony-forming units (cfu) for each colony type.

The colonies were observed using a stereoscopic microscope and were classified as *Aspergillus*, *Penicillium*, *Cladosporium*, yeasts and others. Based on these classified colony counts, the total numbers of fungal colonies per 1 g house dust were calculated.

### 2.3. DNA Extraction, PCR Amplification, Library Preparation and Sequencing

A 200-µL aliquot of the PBS was subjected to DNA extraction using NucleoSpin Soil (Takara Bio., Inc., Shiga, Japan) according to the manufacturer’s instructions.

For the amplification of the internal transcribed spacer 1 (ITS1) region, using polymerase chain reaction (PCR), the ITS1-F_KYO1 primer (5′-CTHGGTCATTTAGAGGAASTAA-3′) and ITS2-KYO2 primer (5′-TTYRCTRCGTTCTTCATC-3′) were used [14]. The PCR program was as follows: 3-min of initial denaturation at 98 °C; 25 cycles of denaturation, at 98 °C for 20 s; annealing at 50 °C for 30 s; and extension at 72 °C for 30 s followed by a final 5 min extension at 72 °C. The PCR reactions were conducted in duplicate. PCR products were purified using Agencourt AMPure XP (Beckman Coulter, Inc., Brea, CA, USA). To conduct tagmentation and for sequencing via the Illumina platform, an adapter sequence was added to the purified PCR products using the following PCR program: 3 min of initial denaturation at 95 °C; eight cycles of denaturation at 95 °C for 30 s; annealing at 55 °C for 30 s; and extension at 72 °C for 30 s followed by a final 5 min of extension at 72 °C. The tagmented libraries were purified again using Agencourt AMPure XP. Sequencing was conducted using the Illumina MiSeq platform with the MiSeq reagent kit v3 (600 cycles). This procedure was conducted commercially by Fasmac Co., Ltd. (Kanagawa, Japan). The read data obtained in the present study were deposited in the DNA Data Bank of Japan under the accession numbers DRR228689–DRR228706.

### 2.4. Sequencing Data Analysis

The primer sequences were trimmed by Cutadapt v1.18 [15]. Quality filtering and trimming were conducted using the dada2 denoise-paired function implemented in Qiime2 [16] with the following parameters: truncLen = 210 and 177 bp for forward and reverse sequence reads, respectively. Dereplication, error correction and read pair merging were conducted using the default settings. The obtained amplified sequence variants (ASVs) were subjected to the following taxonomic assignment.

For the taxonomic assignment of ASVs, we conducted two-step classifications. In the first step, the intact UNITE + INSDC database [17] was used. The BLASTN algorithm was used to conduct taxonomic classification, and the classification of the top hit with an *e*-value of ≤10^−5^ and identity of ≥98% was accepted as the classification of each ASV. In the second step, we used the reads that were classified as fungi in the first step and the modified UNITE + INSDC database. The detailed modifications applied to the database are described as follows: First, we removed database sequences that were not classified at the genus level. Second, we relabeled the sequences classified as *Aspergillus halophilicus*, *Aspergillus montevidensis*, *Aspergillus amstelodami* and *Aspergillus ruber* to *Eurotium halophilicus*, *Eurotium montevidensis*, *Eurotium amstelodami* and *Eurotium ruber*, respectively, because we wanted to use the classifications for sexual generation in this taxon. Additionally, in many cases, the genus *Eurotium* have been observed to be the dominant genus in house dust samples based on the culture method. Therefore, we must distinguish *Eurotium* from *Aspergillus* as the major allergen. The BLASTN algorithm was also used in the second classification step, and the threshold used for classification was the same as that during the first classification step.

### 2.5. Alpha and Beta Diversity Analysis

The obtained ASVs were also subjected to alpha and beta diversity analyses. For the alpha diversity analysis, fungi-assigned ASVs and reads were subjected to a plotting rarefaction curve. For the beta diversity analysis, the dissimilarity among all samples was calculated using the Bray–Curtis dissimilarity metric implemented in Qiime2. After this calculation, all samples were clustered using the unweighted pair group method with arithmetic means (UPGMA).

### 2.6. Calculating the Correlation between the Culture and NGS Methods

Spearman’s Rank correlation coefficient between the culture and NGS was calculated for the genera *Aspergillus*, *Penicillium*, *Cladosporium* and yeast. The colony counts of these four groups, out of all fungal colonies during the culture method, were expressed as a percentage for correlation analysis. Based on the NGS method, the sum of *Aspergillus* and *Eurotium* was considered *Aspergillus*; the sum of *Cladosporium*, *Davidiella* and *Toxicocladosporium* was considered *Cladosporium* and the sum of all yeast species detected in our samples, including *Candida, Cryptococcus, Cystobasidium, Cystofilobasidium, Debaryomyces, Filobasidium, Hanseniaspora, Knufia, Malassezia, Meyerozyma, Pichia, Rhodotorula, Sporobolomyces, Sterigmatomyces* and *Vishniacozyma*, was considered yeasts. The average abundance for each group between the two PCR duplicates was used for correlation analysis. We divided our dust samples into two groups (≥10^6^ cfu/1 g house dust and <10^6^ cfu/1 g house dust) according to the total fungal count determined by the culture method and conducted comparisons between these two groups.

## 3. Results and Discussion

### 3.1. Sampling Results for the NGS Method

In this analysis, approximately 120,000–620,000 reads per sample were obtained (Supplementary Table S1). From each sample, approximately 30,000–330,000 non-chimeric reads were detected. During the first classification step, in most of the PCR duplicates, more than 40% of reads were assigned to fungi. The second-most-common kingdom to which other reads were assigned was Viridiplantae. The sum of reads assigned to fungi, Viridiplantae and unassigned was more than 70% of all non-chimeric sequences in all PCR duplicates.

The non-chimeric reads in samples SEH32-1 and SEH32-2 indicated relatively small numbers of sequencing reads compared to the input reads (Supplementary Table S1). The percentages of non-chimeric reads for SEH32-1 and SEH32-2 were 22% and 25% of input reads, respectively. These results suggested that some fungi may have ITS1 sequences that are longer than 380 bp, which is consistent with the result of a previous study, which reported the distributions of ITS1 lengths in fungi [18]. This previous study reported that the distribution of ITS1 lengths ranges from 100 to 800 bp. In the present study, we targeted only those ITS1 sequences within the range of approximately 200–380 bp because most fungal ITS1 sequences fall within this range. Future iterations of the NGS method should consider fungi with longer ITS1 sequences.

**Figure 1:**
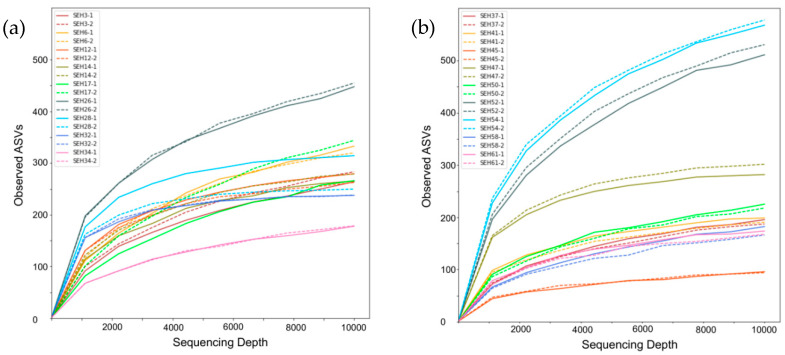
Rarefaction curves of all samples. (**a**) Rarefaction curves for SEH3 to SEH34 and (**b**) rarefaction curves for SEH37 to SEH61. Dotted lines are the duplicates of solid line samples in various colors.

The ASVs constructed by fungi-assigned reads varied from 50–600 among the samples (Figure 1). In contrast, the right ends of the rarefaction curves plateaued, which suggests that 10,000 fungi-assigned reads provide an adequate number of reads for the comprehensive assessment of the fungal communities in house dust samples. In SEH17 and SEH28, the numbers of ASVs were slightly different among PCR duplicates. This difference may stem from by-chance PCR amplification in low DNA density samples or high proportions of unidentified fungi. Based on this result, approximately 15 Mb of data per sample is sufficient to analyze the fungal community in house dust samples. The Illumina MiSeq NGS platform that was used in the present study produces 13–15 Gb in each run (less than 56 h), which would allow approximately 1000 samples to be analyzed in a single run. This estimation suggests that the NGS method can process a much larger number of samples than the culture method in 1 week, even when considering the time necessary for DNA extraction and read analysis. This estimation also implies that other molecular methods, such as DGGE and qPCR, would find it difficult to achieve such numbers of samples along with the amount of sequence variants.

### 3.2. The Robustness of the PCR Step during the NGS Method

**Figure 2:**
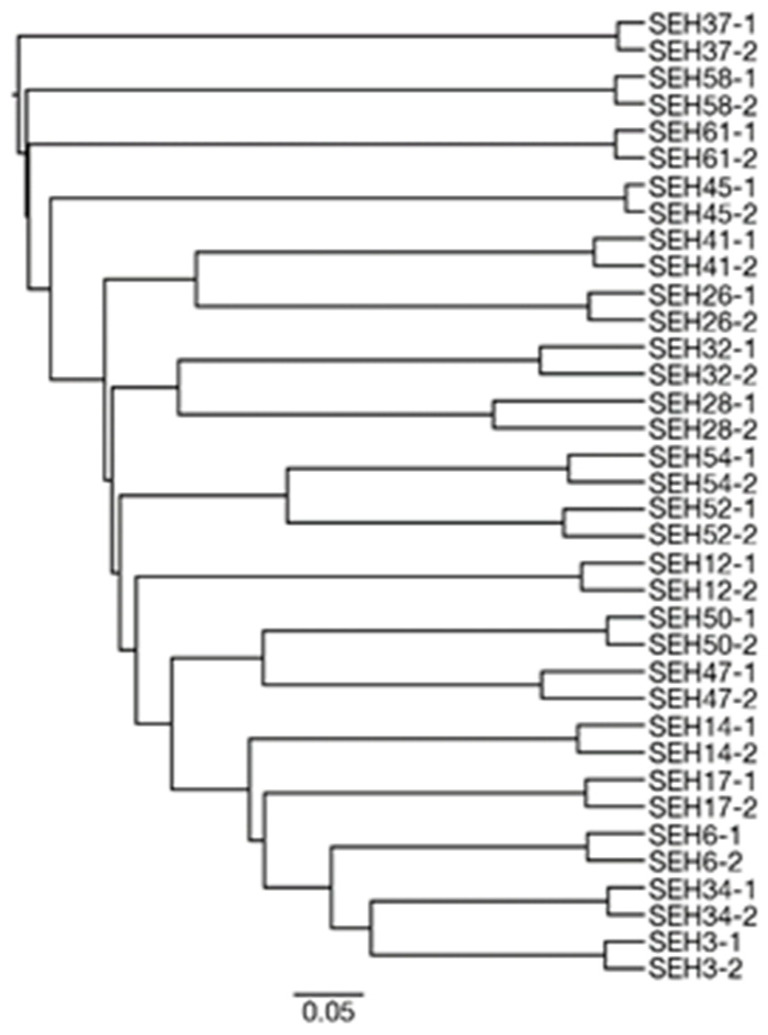
Dendrogram of all samples according to the unweighted pair group method with arithmetic means (UPGMA) based on the beta diversity of community structures.

The PCR step represents a necessary step in this NGS method. During the PCR step, the amount of ITS1 sequence that is amplified can differ widely, even between PCR replications of the same sample because DNA samples derived from house dust are often low in density, and the PCR amplification occurs only in those sequences in which the primers randomly anneal. To confirm the robustness of the PCR step, we compared the beta diversity of fungi-assigned ASVs derived from the PCR duplicates (Figure 2). This result showed that the community structures of the PCR duplicates from each sample were more closely related than those of other duplicates from other samples. This result indicates that variations between PCR duplicates from the same sample were smaller than those between samples. Hence, the PCR step used in the present study can be considered robust for the fungal community analysis of house dust samples.

### 3.3. Fungal Community Structure Comparison between the Culture and NGS Methods

**Figure 3:**
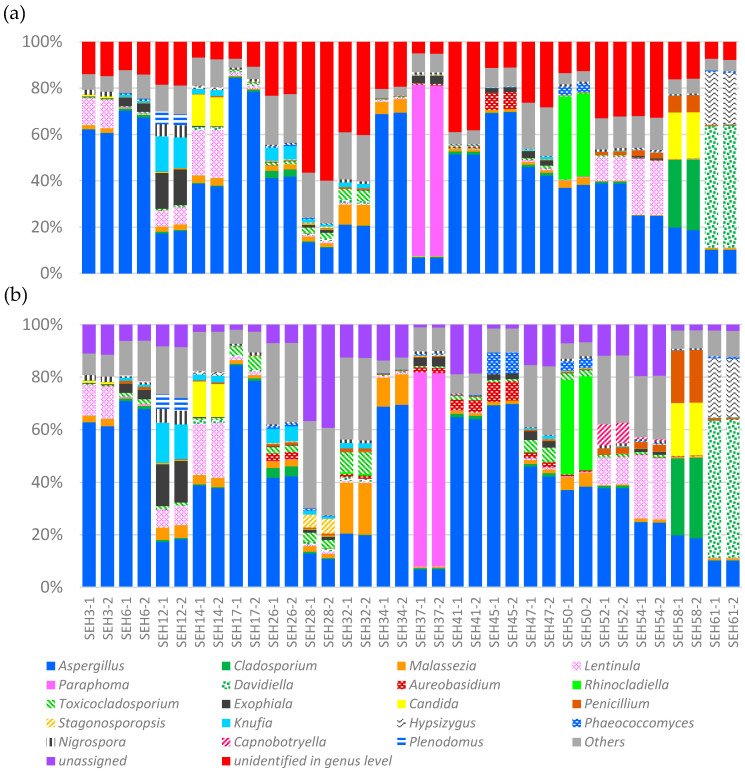
Taxonomic compositions of the fungal communities in 36 PCR duplicates of house dust samples. (**a**) Without the removal of database sequences classified at the genus level; (**b**) with those sequences removed. Gray bar indicates the ‘Others’ category defined as the occupancy rate of the total remaining species other than the 19 genera shown in this figure. Red bar indicates the ‘unidentified at the genus-level category’ defined as the assignment to database registrations that are not classified at the genus level. Purple bar indicates the ‘unassigned’ category defined as the failure to be assigned to any database sequences with more than a 98% homology.

We conducted a taxonomic assignment of ASVs at the genus level during the second classification step. Here, we show 19 genera that represent more than 5.0% of each sample using bar plots (Figure 3). The ‘unidentified at the genus-level category’ in Figure 3a is defined as the assignment to database registrations that are not classified at the genus level and the ‘unassigned’ category in Figure 3b is defined as the failure to be assigned to any database sequences with more than 98% homology. The ‘Others’ category is defined as the occupancy rate of the total remaining species other than the 19 genera shown in Figure 3.

During the first classification step, we conducted the taxonomic assignment of ASVs using the intact UNITE + INSDC database, which includes those sequences that were not classified at the genus level. The results—as indicated in Figure 3a—show the fungal genus-level taxonomic compositions from the first step. The results of the second classification step are presented in Figure 3b. We could detect the major taxa, such as *Aspergillus*, *Penicillium* and *Cladosporium*, that are recognized as relatively high-abundance genera in taxonomic compositions using the culture method. In our NGS method, the average and maximum relative abundance values for these three genera, among 36 PCR libraries, were 40.3% and 84.5% for *Aspergillus*, 1.8% and 20.1% for *Penicillium* and 2.3% and 30.7% for *Cladosporium*. Furthermore, we detected a high abundance of *Malassezia* in all samples. This genus is very important for human health because it includes the causative species for skin diseases in humans [19]. *Malassezia* only grows in a special medium using the culture method because of lipid requirements; therefore, using universal media and the culture method often fails to detect this genus in samples. The relatively easy confirmation of the presence or absence of this genus in a sample using NGS, with time and effort similar to that for other easily culturable genera, is notable.

The differences in the occupancy rates between Figure 3a,b for each genus and others are summarized in Table 1. Furthermore, the degree of change in the occupancy rates between unassigned reads, with and without genus-level classifications, was calculated as the difference between ‘unassigned’ in Figure 3b and ‘unidentified at the genus level’ in Figure 3a. Our results indicate the decreasing occupancy rates of unassigned reads with genus-level classifications in all house dust samples in the present study. When comparing the differences in the assignment rates for each of the 19 genera, the genera *Malassezia* and *Toxicocladosporium* had the highest increase in assigned reads. The reads belonging to the genera *Capnobotryella* and *Stagonosporopsis* were not assigned at all when the analysis was conducted using an intact database; however, these genera were detected in several PCR libraries with maximum rates of 8.2% and 5.7%, respectively, after modifying the database (Figure 3b). Moreover, the ‘Others’ rates with the modified database increased compared with the rates when using the intact database for all samples.

The proportions of reads assigned to particular genera increased after modifying the database because non-culture-based methods, including NGS, have returned many sequences that are registered as an ‘uncultured clone’ in recent years, which are mostly not classified. Especially because the genera *Malassezia* and *Toxicocladosporium* are distributed over various environments, when the registered sequences were fully examined for genus-level identification, many of these uncultured clone sequences have been assumed to belong to these two genera. In this situation and without sufficient data cleaning to remove uncultured clone sequences that have not been sufficiently classified, sequence reads are often assigned to these clones based on high sequence homology. However, we believe that the removal of uncultured clone sequences that have not been classified at the genus level allow our reads to hit the registered sequences of particular genera, including *Malassezia*, *Toxicocladosporium*, *Capnobotryella* and *Stagonosporopsis*, as the ‘second homological’ sequence.

These results suggest that the removal of database registrations that have not been sufficiently classified facilitates the increased frequency of read-assignments to particular genera—including minority genera—which improved the accuracy and increased the understanding of the mycoflora populations in the samples. This suggests that this step is necessary to obtain meaningful analysis results.

In addition to the analysis shown in Figure 3, we verified the occupancy rates of 22 genera [13], which have often been detected in previous surveys of fungal flora, using the culture method (Supplementary Table S2). We found that five genera, *Aspergillus* (maximum: 84.2%), *Aureobasidium* (14.4%), *Candida* (12.2%), *Cladosporium* (46.3%) and *Penicillium* (16.0%), had occupancy rates of at least 5% in at least one house dust sample. These genera, which were detected at occupancy rates of at least 5% in the present study, are known to be detected at particularly high frequencies and concentrations in living environments when using the culture method [8], indicating that our results are consistent with those obtained using the culture method. We also verified the occupancy rates of 21 genera relevant to human health [20,21,22] (Supplementary Table S3). We found that seven genera, including *Aspergillus* (maximum: 84.2%), *Candida* (12.2%), *Exophiala* (14.1%) *Malassezia* (27.2%), *Alternaria* (1.1%), *Cladophialophora* (4.4%) and *Hortaea* (2.7%), had occupancy rates of at least 1% in at least one house dust sample. All other genera had occupancy rates of less than 1%. Our results suggest that the genus *Aspergillus* is the most important fungi because it is characterized by high pathogenicity and appears at high frequencies and concentrations in dwelling environments. Genera other than *Aspergillus* do not appear with the same high frequency or concentration, although they are still considered major fungal pathogens.

### 3.4. Correlations between the Culture and NGS-based Methods

**Figure 4:**
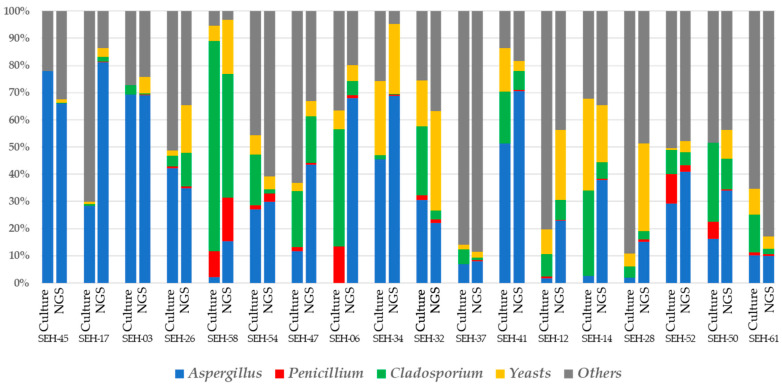
Comparisons of fungal occupancy rates between the next-generation sequencing (NGS) and culture methods. The bars are arranged from left to right by increasing total fungal counts/1 g house dust.

We compared the occupancy rates of each of the four groups based on the results of the culture and NGS methods (Figure 4). Three genera, including *Aspergillus*, *Cladosporium* and *Penicillium* and a group of yeasts—which belong to a major taxon because of a relatively higher abundance than other genera in the taxonomic compositions—are easy to recognize using the culture method. Therefore, these four groups are good markers when we compare currents between the NGS and culture methods. In this figure, the bars are arranged from left to right by increasing total fungal counts/1 g of house dust. Overall, this figure shows that the results obtained using the NGS method corelate with those obtained using the culture method, especially for dust samples SHE-03, 26 and 45, which show the highest correlations with the culture method, although some samples had comparably low correlations, such as SHE-06, 12 and 14.

In each house dust sample, the bar on the left shows the results according to the culture method, whereas the bar on the right shows the results using the NGS method. In this panel, the bars are arranged from left to right by increasing the total fungal counts/1 g in house dust.

To investigate the degree of correlation between the results of the NGS and culture methods, we calculated Spearman’s rank correlation coefficients for *Aspergillus*, *Penicillium, Cladosporium* and yeasts. Furthermore, we divided our dust samples into two groups according to the total fungal count as determined by the culture method depending on whether they had more or less than 10^6^ cfu/1 g house dust and compared the two groups (Table 2). Our results confirmed that the NGS method showed a high correlation with the culture method when the total fungal count of the sample was above 10^6^ cfu/1 g of house dust. However, the correlations between the results of these two methods were weak in several samples. When using the culture method—when a small amount of house dust sample was collected and the total fungal count was low—the colony count per agar plate tended to be smaller, detection varied greatly, and uncertainty increased. Therefore, that culture method resulted in unstable detection in small samples, resulting in a weak correlation. In the present study, samples SHE-6, 12, 14 and 50, which showed relatively weak correlations between methods, had low sample weights and a total fungal count/1 g of house dust of 10^6^ cfu or less. Therefore, they were classified in the low group. Moreover, when the total fungal count was high in the fungal flora analysis experiment, using the culture method, the serial dilution method was applied so that the total fungal count per agar plate ranged from approximately 30 to 80, which increased the labor required for the experiment. Because the NGS method showed a favorable correlation with the culture method, especially for high fungal count samples, the NGS method can likely be used instead and with high reliability to reduce the labor required for experiments.

## 4. Conclusions

In the present study, we applied the NGS method to the fungal community analysis of house dust and showed improvements in the comprehensiveness and the time and labor costs over those for the culture method. The improved comprehensiveness also revealed that some genera, including those that are disease-causative, especially as allergens, have been overlooked by the culture method, but can be found in human living spaces at relatively high percentages of the community, such as the genus *Malassezia* and including minority genera, such as the genus *Schizophyllum* [20], which can be difficult to detect using the culture method. These results show the usefulness of the NGS method in indoor environments and will promote the application of the NGS method as the one of major approaches to investigate the fungal communities in indoor environments. However, the present study also indicates that the easily available databases require additional modifications—including the removal of registrations—that have not been sufficiently classified at the genus level, to facilitate more efficient identification of genera. Moreover, the present NGS method can be difficult for quantitative assessment and the detection of species that have not yet been registered in the database is impossible. Improved techniques are necessary and additional registrations with sufficient levels of taxonomic information, must be added to the database. In the near future, the NGS method will provide insights regarding the effects of the abundance, diversity and composition of indoor mycoflora depending on climate and geographical conditions, dwelling performance and resident living styles to achieve healthy living spaces with reduced levels of fungal allergies, which cannot be sufficiently analyzed using the culture technique.

## Figures and Tables

**Table 1:** Comparison of fungal occupancy rates between the use of the intact and modified database.

House Dust Sample	Rate Difference between with Intact DB and with Modified DB *^1^(%)			
	Unassigned Reads with Genus Level Classification *^2^	Other Genera in Figure 3	Maximum in a Sample	
			Genus	Difference (%)
SHE-3	−3.1	1.5	*Malassezia*	0.8
SHE-6	−7.0	4.2	*Toxicocladosporium*	1.2
SHE-12	−10.3	6.9	*Malassezia*	2.5
SHE-14	−4.4	3.0	*Toxicocladosporium*	0.6
SHE-17	−6.7	2.1	*Toxicocladosporium*	3.5
SHE-26	−15.9	9.4	*Aureobasidium*	2.7
SHE-28	−20.2	14.4	*Stagonosporopsis*	4.8
SHE-32	−27.1	11.2	*Malassezia*	10.7
SHE-34	−6.8	0.9	*Malassezia*	5.8
SHE-37	−4.0	0.9	*Aureobasidium*	1.4
SHE-41	−19.8	2.2	*Aspergillus*	13.0
SHE-45	−9.6	0.7	*Phaeococcomyces*	7.9
SHE-47	−11.6	4.4	*Toxicocladosporium*	4.0
SHE-50	−6.2	0.9	*Toxicocladosporium*	2.2
SHE-52	−20.8	12.3	*Capnobotryella*	8.0
SHE-54	−12.9	9.9	*Malassezia*	1.0
SHE-58	−13.9	0.5	*Penicillium*	12.8
SHE-61	−5.2	4.9	*Penicillium*	0.1

*^1^ (Intact UNITE+INSDC)-(Modified UNITE+INSDC) *^2^ (“unassigned” in Figure 3b)-(“unidentified in the genus level” in Figure 3a).

**Table 2:** Spearman’s rank correlation coefficient between the NGS and culture methods and a comparison of the two groups of house dust samples as divided by whether the total fungal counts/1 g house dust were above or below 10^6^ colony-forming units (cfu).

Fungal Group	Correlation Coefficients	
	Total Fungal Count <10^6^ cfu	Total Fungal Count >10^6^ cfu
*Aspergillus*	0.29	0.66
*Penicillium*	0.57	0.94
*Cladosporium*	0.38	0.37
Yeasts	0.41	0.46

## Supplementary Materials

The following are available online at https://www.mdpi.com/1660-4601/17/16/5842/s1, Table S1: Numbers of analyzed and assigned reads, following the removal of database sequences not classified at the genus level, Table S2: The relative abundance of fungi that are often observed in culture method., Table S3: The relative abundance of medically important fungi.
